# Mutualism and Adaptive Divergence: Co-Invasion of a Heterogeneous Grassland by an Exotic Legume-Rhizobium Symbiosis

**DOI:** 10.1371/journal.pone.0027935

**Published:** 2011-12-09

**Authors:** Stephanie S. Porter, Maureen L. Stanton, Kevin J. Rice

**Affiliations:** 1 Section of Evolution and Ecology, University of California Davis, Davis, California, United States of America; 2 Center for Population Biology, University of California Davis, Davis, California, United States of America; 3 Department of Plant Sciences, University of California Davis, Davis, California, United States of America; University of Tartu, Estonia

## Abstract

Species interactions play a critical role in biological invasions. For example, exotic plant and microbe mutualists can facilitate each other's spread as they co-invade novel ranges. Environmental context may influence the effect of mutualisms on invasions in heterogeneous environments, however these effects are poorly understood. We examined the mutualism between the legume, *Medicago polymorpha*, and the rhizobium, *Ensifer medicae*, which have both invaded California grasslands. Many of these invaded grasslands are composed of a patchwork of harsh serpentine and relatively benign non-serpentine soils. We grew legume genotypes collected from serpentine or non-serpentine soil in both types of soil in combination with rhizobium genotypes from serpentine or non-serpentine soils and in the absence of rhizobia. Legumes invested more strongly in the mutualism in the home soil type and trends in fitness suggested that this ecotypic divergence was adaptive. Serpentine legumes had greater allocation to symbiotic root nodules in serpentine soil than did non-serpentine legumes and non-serpentine legumes had greater allocation to nodules in non-serpentine soil than did serpentine legumes. Therefore, this invasive legume has undergone the rapid evolution of divergence for soil-specific investment in the mutualism. Contrary to theoretical expectations, the mutualism was less beneficial for legumes grown on the stressful serpentine soil than on the non-serpentine soil, possibly due to the inhibitory effects of serpentine on the benefits derived from the interaction. The soil-specific ability to allocate to a robust microbial mutualism may be a critical, and previously overlooked, adaptation for plants adapting to heterogeneous environments during invasion.

## Introduction

Species inevitably experience heterogeneity in abiotic and biotic environments across their distributions. Heterogeneous selection resulting from these differences can drive patterns of adaptive differentiation or local adaptation under the right balance of gene flow, heritable variation and genetic constraint [1], [2], [3]. Research on adaptive divergence in plant populations that span both harsh, low-nutrient serpentine soil and more benign non-serpentine soil, has provided classic examples of adaptive divergence as a means for a single species to persist across striking environmental heterogeneity [4], [5], [6]. Adaptive divergence in large native plant populations is relatively common [7]. However, it is less clear whether adaptive divergence plays as large a role in the evolutionary dynamics of invasive species, for which local patterns of selection are relatively novel. Some invasives show evidence of adaptive divergence across novel environmental heterogeneity, while others are generalists that rely upon plasticity to maintain high fitness across environments [4], [8], [9], [10], [11]. For species that co-invade environments as tightly inter-connected mutualists, partners might undergo genetic specialization or generalization to different degrees, depending in part on relative rates of gene flow and selection [12].

Mutualisms are major drivers of ecological and evolutionary processes and can be important promoters of plant invasion [13], [14], [15], [16]. There is a growing appreciation for the importance of such positive biotic interactions for invasion dynamics; however we have a weak grasp of the role of adaptive divergence in invasive mutualisms that span heterogeneous environments. Because partners are tightly inter-dependent in symbiotic mutualisms, a microevolutionary shift in one partner due to abiotic heterogeneity may cause a concomitant shift in biotic selection on the other [17]. Heterogeneity in abiotic conditions across environments can affect the strength of selection, generating mosaics in the outcome of the mutualism for either partner [18]. For example, mutualisms are predicted to be more mutually beneficial in low-quality environments [19], [20], [21], [22] and of increased importance to invasion of these environments. Examination of positive interactions between plants and soil microbes has yielded insight into both the ecological dynamics of symbiotic mutualism in invasive species [23] and the process of adaptive divergence across habitats in long-established native symbiotic mutualisms [21]. While many plants that rely on symbiotic mutualisms are invasive across heterogeneous environments, little research has examined simultaneously the importance of adaptive divergence in both co-invading partners colonizing heterogeneous landscapes.

The legume-rhizobium symbiosis is an important system for such research because it includes examples of successful invaders that can have strong community and ecosystem-level effects [24], [25], as well as examples of adaptive divergence across heterogeneous environments for both symbiotic partners [7], [26], [27]. Legumes provide photosynthates and a root nodule environment to their rhizobial symbionts, while rhizobia are capable of fixing atmospheric dinitrogen and delivering it as ammonium to the plant. The symbiosis is horizontally transmitted and rhizobia persist as free-living bacteria in the soil between legume generations (reviewed in [28], [29]). Most successful legume invaders have high levels of nodulation in the invaded range, indicating that the mutualism is functional and ubiquitous during spread [16], [30]. Legume invaders display a range of specificity for their rhizobial mutualists. Many legumes are generalists that can associate with diverse taxa of native rhizobia while others are specialists that associate with narrower pools of native rhizobia symbiotic with related native legumes [31], [32]. Both these classes of invaders tap into existing populations of native rhizobia that are pre-adapted to local edaphic conditions [14], [33]. Less well documented, though possibly no less common, are co-invading legumes and rhizobia that are both exotic [34], [35]. Exotic rhizobia can face strong challenges to establishment in novel habitats, including abiotic stresses in the soil, competition with other soil biota, and securing access to host legumes at adequate density to maintain viable soil populations.

Here, we examine the symbiosis between the legume *Medicago polymorpha* L. (Burr Medic) and the rhizobium *Ensifer medicae* [36]. These two species of Eurasian and African Mediterranean origin have widely invaded grasslands in North America, South America and Australia [37]. *Medicago polymorpha* was well-established in Mexico by the late 1700's [38], and invaded California in the late 1800's [39]. *Medicago polymorpha* forms effective root nodules almost exclusively with *E. medicae* in both the native and invaded range [40] and is therefore considered notably specialized on this rhizobium species [41]. Because *E. medicae* associates naturally with legumes of the genus *Medicago*, none of which are native to the New World [37], *E. medicae* is presumed to have been introduced to the New World along with its host. In California, these mutualists have successfully invaded heterogeneous grassland habitats containing both physiologically harsh, low-nutrient serpentine soil as well as more benign non-serpentine soils [42], [43].

We examine whether this invasive legume-rhizobium symbiosis exhibits adaptive ecotypic divergence across the novel soil matrix and assess the role mutualistic traits may play in potential adaptive divergence in a multi-part study: 1) To understand how plants may diverge phenotypically, we examine whether plants show variation in reproductive, symbiotic or vegetative traits consistent with adaptive ecotypic divergence across soil types. 2) We investigate these same traits to determine whether plants show adaptive divergence in their association with rhizobia from contrasting soils. 3) Because the process of co-invasion depends on fitness outcomes for both mutualist partners, we also examine whether rhizobium symbiotic fitness shows similar trends in adaptive divergence. 4) To determine how important the mutualism is to fitness components that contribute to the invasiveness of *M. polymorpha*, we examine whether vegetative traits and reproductive output are equally affected by the mutualism and whether the amount of benefit the mutualism provides to the plant is greater in nutrient-poor serpentine soil. 5) Finally, we examine whether inoculating plants with single strains of rhizobia or mixes of numerous strains affects the outcome of the above tests.

## Methods

### Collections

Lineages of *M. polymorpha* and *E. medicae* were collected at persistent populations of *M. polymorpha* from four serpentine and three non-serpentine sites (Fig. S1, Table S1), that have been characterized for over a decade [42], [43] at the University of California's Donald and Sylvia McLaughlin Natural Reserve (38°52′26″N; 122°25′54″W). Mature seed pods and root nodules harboring rhizobia were collected from plants in the field. Seed was bulked at the level of population, but each seed pod was collected from a different maternal plant, with an average of 25 maternal families per population.

Field soil was collected in reserve grasslands that were not the home sites for the above populations. This allowed us to test representative serpentine and non-serpentine soils without providing plant or rhizobial lineages with a potential home-site advantage. Serpentine soil was collected from a representative serpentine outcrop and non-serpentine soil was collected adjacent to an oak woodland (Fig. S1, Table S1). Soils at the reserve are thin and have low fertility. The reserve's serpentine soil is deficient in nitrogen, phosphorous and calcium and enriched in magnesium and nickel, relative to the non-serpentine soils [43], [44]. Each soil type was collected dry, sifted through 1-cm gauge mesh, and thoroughly mixed. A 1∶1 mix by volume of field soil and inert silica sand was created to avoid compaction of the sifted soil in greenhouse conditions. This mixture was thoroughly steam pasteurized before the experiment. Therefore, the only source of rhizobia in the experiment was inoculation. Plants that did not receive a rhizobial inoculation formed no nodules.

### Isolation of rhizobia

It is widely assumed that *M. polymorpha* displays a high specificity for *E. medicae* rhizobia throughout its native and invaded range. However, the rhizobia associated with *M. polymorpha* on serpentine soils had not previously been characterized. We sampled 180 intact plants (90 from three serpentine and 90 from three non-serpentine populations), with nodules attached to the roots. One nodule isolate from a randomly selected nodule was extracted per plant using standard axenic culturing technique [45] and three restreakings from single colonies and preserved in glycerol at −80°C. The culturing technique therefore required numerous generations of growth in a common laboratory environment before the experiment. We utlilized DNA sequencing of the 16S ribosomal DNA and FUMC intergenic region [46] for all isolates. BLAST searches of all the above loci in Genbank indicated that nearly all (>95%) of the sequences were closer to *Ensifer medicae* than to other rhizobia, including sister species *Ensifer meliloti.* In this way we verified that *E. medicae* is the rhizobial partner of *M. polymorpha* throughout the McLaughlin Natural Reserve (Porter, unpub. ms).

### Experimental Design

Bulked seed from four serpentine and three non-serpentine plant populations were used in a factorial design, paired with rhizobia from serpentine or non-serpentine origins, and planted in either serpentine or non-serpentine field soil. The experiment was arranged as a split-split plot design (Fig. S2, Table S2). The main plot occurred at the level of rack, with each rack containing 26 plants grown in 66 ml Cone-Tainers (Stewe & Sons Inc, Tangent OR). Rhizobium treatment was applied at the level of a rack to avoid cross contamination between plants sharing a rack. The split plot occurred at the level of a half-rack, within which 13 plants were bottom-watered in a common tray. Field soil treatment was applied at the level of half-rack to avoid cross-leaching of field soil solutes. The split-split plot occurred within each half rack where plants were either of serpentine or non-serpentine origin, arranged in a complete randomized design within the half-rack. Two plants from each plant population were grown in each half rack. A single plant per half rack was used for one serpentine population for which fewer seeds were available (Fig. S2).

Rhizobium inoculation consisted of applying ∼2×10^5^ rhizobium cells in 0.5 mL of water to the soil at the base of each plant. From the above populations, two serpentine (S1 and S2) and two non-serpentine (N1 and N2) *E. medicae* isolates were randomly selected and then applied as single-isolate inocula to two racks each, for a total of eight single-isolate racks. Two mixed-isolate inocula were composed of nine randomly selected *E. medicae* isolates from either serpentine (Smix) or non-serpentine (Nmix) sites, and applied to four racks each, for a total of eight mixed-isolate racks. Two racks were inoculated with water and grown with no rhizobia. These plants served as a methodological control for rhizobial cross-contamination and allowed measurements of plant traits in the absence of rhizobia. No nodules were detected on the roots collected from control plants, indicating no or very low levels of cross-contamination of rhizobia occurred in this experiment. Control racks contained 18 plants per half-rack, a comparable density to the inoculated plants. Thus, 416 plants were grown with rhizobia and 72 were grown without rhizobia (404 and 70 plants survived to maturity, respectively). Racks were placed in a randomized order in the greenhouse and were re-randomized monthly to minimize the effects of environmental heterogeneity in the greenhouse.

### Germination

Each seed was individually weighed, scarified with a razor, and surface-sterilized in full strength commercial bleach (3% sodium hypochlorite) for 1.5 minutes, followed by eight rinses of sterile water. Seeds were imbibed and vernalized at 4°C in the dark for eight days. Germinating seeds were planted into Cone-Tainers containing rhizobium-free steam-pasteurized soil. At the appearance of the first trifoliate leaf, plants were inoculated with either rhizobia or water without rhizobia as controls.

### Inoculation

*Ensifer medicae* cultures were grown in liquid tryptone-yeast media for 48 hours at 30°C at 300 rpm. Immediately before inoculating plants, inocula were centrifuged and re-suspended to 4×10^5^ cells mL^−1^ in water (based on OD_600_). For mixed isolate inocula, equal volumes of each of the nine component isolates were combined. Each plant received 0.5 ml of inoculum, only one type of inoculum was used per rack, and racks were spaced 30 cm apart to avoid cross-contamination. Plants were bottom-watered regularly with reverse-osmosis purified water; so, their only source of nutrients was derived from the treatment field soil and rhizobia.

### Trait measurements

Plants were grown for 90 days to maturity during the natural California winter annual growing season in a greenhouse. At harvest, belowground tissue was washed and frozen. Samples were subsequently thawed and nodules were counted and separated. Root and nodule tissue were then dried to a constant weight at 60°C and weighed (mg). Pods were counted, dried and crushed to separate seeds, which were then weighed (mg). Plant reproduction was measured as the number of pods produced and total weight of seed produced. Number of pods is a key fitness trait for this plant because seeds are usually retained within the tough, spiraled burr as a dispersal unit and germinate through the pod. Total length of stem tissue was measured (cm) to estimate above-ground plant size; root weight was used as an estimate of below-ground plant size. Plant symbiotic investment was measured as the number of nodules produced and the total weight of nodules produced. Nodule number and mass are also an important component of rhizobial fitness and were therefore used to estimate the fitness of rhizobia in symbiosis [47], [48]. Over the course of the experiment 5% of the individual trait values for the 474 plants that survived to maturity were reported as missing due to human error. This loss of data was small and haphazard and therefore unlikely to bias analyses.

### Analysis

A mixed model analysis of co-variance (ANCOVA in the MIXED procedure, SAS Institute, 2006) was used in a split-split plot structure to test whether reproductive, vegetative and symbiotic traits varied among plant soil origin genotypes, rhizobium soil origin genotypes or destination soil types. To focus on the effects associated with symbiotic genotype combinations, only inoculated plants were used in the first analysis and a separate simplified model was used to compare inoculated vs. uninoculated plants.

Reproductive, vegetative and symbiotic traits for symbiotic plants were evaluated in separate ANCOVAs with the fixed effects listed in Table 1. Plants and rhizobia were either of serpentine or non-serpentine origin. Both soil types had single and mixed rhizobium diversity treatments and each rhizobium diversity treatment contained one or two rhizobium identity treatments. Therefore rhizobium diversity was nested within rhizobium origin and rhizobium identity was nested within rhizobia diversity. The initial weight of each seed was included as a covariate in the analysis to adjust the model for variable maternal investment. To control for spatial heterogeneity in the greenhouse, rack was included as a random effect and was nested within rhizobium origin soil, rhizobium identity and rhizobium diversity, because these treatments were imposed at the level of rack. The following random effects were automatically applied by Proc Mixed as error terms in the split-split plot design: rack for the main plot and destination soil by rack for the split plot. Because the focus of the experiment was to test for ecotypic adaptive divergence at the level of soil type, responses were averaged within each plant population of origin for a given half-rack, which helped to stabilize mean responses. This was accomplished by including the destination soil by plant origin by rack interaction as a random effect and leaving plant population out of the model, allowing populations to serve as replicates of plant origin soil, without themselves being the subject of the analysis. This error term also allowed PROC MIXED to adjust for unequal replication and variance of these population means. Raw data were transformed by either a natural log or square root transformation to meet the assumptions of normality and homogeneity of variance. Each class of plant traits was examined by testing for experimental effects on two response variables: pod number and seed weight for reproductive traits, stem length and root weight for vegetative traits, and nodule number and nodule mass for symbiotic traits. Therefore the alpha level cut-off for significance was Bonferroni-corrected to 0.025 to account for two independent tests per trait class. Where main or interactive fixed effects in the model were significant, least squares mean comparisons (LSmeans) were used to evaluate the significance and direction of effects within a treatment.

**Table 1:** Mixed model ANCOVA for *M. polymorpha* and *E. medicae* isolates from non-serpentine and serpentine soil origins grown together in non-serpentine and serpentine destination soils in the greenhouse^1^.

MIXED MODEL ANCOVA			F-STATISTICS					
	df		Reproductive traits		Vegetative traits		Symbiotic traits	
Fixed effects	n	d	Ln pod num	Sqrt seed mass	Ln stem length	Ln root mass	Ln nodule num	Ln nodule mass
P origin	1	31.3	4.6 †	**9.05** *b*	**42.7** *d*	**6.69** *a*	0.3	0.65
Dest soil	1	14.4	**52.1** *d*	**102.9** *d*	**40.9** *d*	2.54	1.34	3.26
R origin	1	9.7	0.01	0.16	0.92	0.31	**8.35** *a*	**11.5** *b*
R div (R origin)	2	9.8	0.13	0.1	0.57	2.36	1.19	**7.79** *a*
R ID (R origin * R div)	2	9.5	0.01	0.09	0.08	0.01	0.57	3.07
P origin * Dest soil	1	25.0	**10.1** *b*	5.19†	2.47	4.43†	5.63†	**22.7** *d*
P origin * R origin	1	24.8	**13.5** *b*	2.7	1.99	1.56	1.55	1.52
P origin * R div	2	25.1	1.81	1.34	0.53	0.11	1.1	0.8
P origin * R ID (R origin * R div)	2	23.8	2.36	0.21	0.27	1.76	0.65	0.26
R origin * dest soil	1	14.2	0.5	0.39	0.64	0.39	0.07	0.16
P origin * R origin * dest soil	1	25.0	0.24	0	0.7	0.03	0.66	0.32
Initial seed weight covariate	1	352.3	2.37	0.4	0.04	0.86	**5.23** *a*	0.56

1 Random effects reflecting the main plot, split-plot and split-split-plot levels of organization of the factorial experimental design were not of experimental interest and are not shown. Bonferroni correction for two hypotheses per test yields alpha = 0.025 as a threshold for significance; F-statistics in bold are significant.

† P<0.05;

*a,* P<0.025; *b,* P<0.01; *c,* P<0.001; *d*, P<0.0001. Abbreviations: P, plant; R, rhizobium; div, diversity; df, degrees of freedom; n, numerator; d, mean denominator; Ln, natural log transform; Sqrt, square-root transform; num, number.

After running this ANCOVA a further post-hoc test was run to clarify whether significant main and interactive effects for nodule weight were driven by allocation to nodule mass independent of allocation to root mass. Here nodule mass was divided by root mass for each plant and the effects of interest were tested in the original ANCOVA model. For this data set transformations of the data improved but did not fully alleviate violations of the assumption of homogeneity of variance. Accordingly, observations were weighted by the inverse of the variance by destination soil to compensate for this violation [49], [50]).

A simplified version of the ANCOVA model (MIXED procedure, SAS Institute, 2006 [51]), in which rhizobium factors were simplified to inoculation status, either inoculated or uninoculated, was applied to both inoculated and uninoculated plants for reproductive and vegetative traits. Here fixed effects consisted of inoculation status, plant origin soil, destination soil, plant origin soil by destination soil, inoculation status by destination soil and inoculation status by plant origin soil. Proc Mixed applied random effects automatically as error terms in the split-split plot design as above. For this data set transformations of the data improved but did not fully alleviate violations of the assumption of homogeneity of variance for weight of seed and weight of roots. Accordingly, these raw observations were weighted by the inverse of the variance for inoculated vs. uninoculated plants to compensate for this violation [49], [50]).

## Results

### 1) Plant adaptive divergence across environments

**Figure 1:**
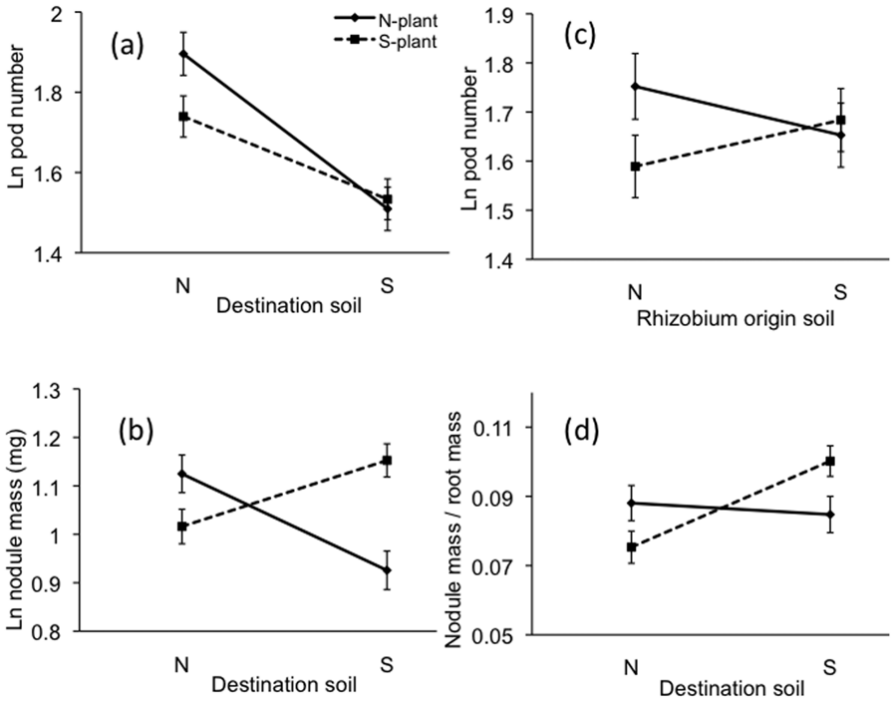
Adaptive differentiation in *Medicago polymorpha* for reproductive and symbiotic traits. Reaction norm plots for plants collected from non-serpentine (N-plant) vs. serpentine (S-plant) soils. (**a**) Number of pods produced in non-serpentine vs. serpentine destination soil. (**b**) Nodule mass produced in non-serpentine vs. serpentine destination soil. (**c**) Number of pods produced when plants were inoculated with rhizobia from non-serpentine vs. serpentine origin (**d**) Nodule mass per mass of root produced in non-serpentine vs. serpentine destination soil. Fitness indices are least square means (LSM) from the ANCOVA analysis of natural log (Ln) transformed values. Bars are +/− LSM standard error.

**Figure 2:**
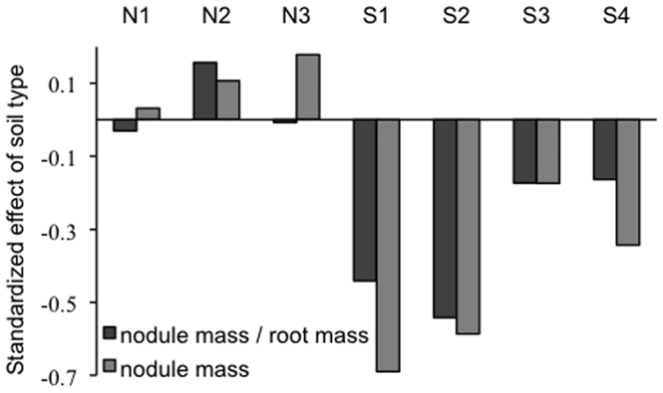
Effect of soil type on plant investment in mutualism. Proportional difference in symbiotic tissue investment for serpentine and non-serpentine plant populations grown in the two soil types. Standardized effect of soil type is calculated as the ((population mean value in non-serpentine soil)-(population mean value in serpentine))/(population mean value in non-serpentine soil). Mean values for each non-serpentine (N1–N3) and serpentine (S1, S2, S3, S4) population are presented for both allocation to biomass of nodule tissue per biomass of root tissue (nodule mass/root mass) and the raw biomass of nodule tissue (nodule mass).

*Medicago polymorpha* exhibited trends for adaptive ecotypic divergence between soil types for reproductive output and symbiotic traits, but similar trends were not statistically significant for vegetative traits. ANCOVA indicated that serpentine and non-serpentine plants allocated differently to reproductive and symbiotic traits when growing in the two destination soil environments (pod number, P<0.01; nodule mass, P<0.0001) (Table 1, Fig. 1a,b). Least squares mean comparisons revealed crossing reaction norms in which non-serpentine origin plants had greater pod output in non-serpentine destination soil than did serpentine origin plants (P<0.001) and serpentine origin plants tended to have higher reproductive output in serpentine destination soil, though this latter pattern was not statistically significant. Seed mass responded similarly to the experimental treatments. Least squares mean comparisons of nodule mass revealed crossing reaction norms, in which non-serpentine origin plants produced greater nodule mass than did serpentine origin plants when growing in non-serpentine destination soil (P<0.001) and serpentine origin plants produced greater nodule mass than did non-serpentine plants when growing in serpentine destination soil (P<0.025) (Fig. 2). Changes in nodule number followed qualitatively similar patterns. Plant ecotype differences in allocation to nodule mass in the destination soil environments were not driven solely by greater belowground biomass in the home soil type. Ecotypes differed in their proportional allocation to nodule biomass per mass of root in the two destination soil environments ((nodule mass)/(root mass); F_1,22_ = 7.98, P<0.01) (Fig. 1d). Least square mean comparisons revealed that serpentine plants had greater allocation to nodule biomass per mass of root than did non-serpentine plants in serpentine soil (P<0.05) and non-serpentine plants exhibited a pattern of greater allocation to nodule biomass per mass of root than did serpentine plants in non-serpentine soil, though this pattern was not significant (Fig. 1d, Fig. 2). Overall, nodule biomass in a plant was positively correlated with pod output (P<0.0001, r = 0.213) and this pattern was driven primarily by plants on non-serpentine destination soil (non-serpentine destination soil: P<0.0001, r = 0.421; serpentine destination soil: P = 0.115, r = 0.114). Differences in the effects of destination soil on the plant ecotypes were statistically significant and stronger for plant reproductive and symbiotic traits than they were for vegetative traits, where patterns were not significant, though root weight showed a qualitatively similar marginal pattern (Table 1).

Non-serpentine soil was consistently a higher quality environment for symbiotic plants, yielding higher plant reproductive output (pod number, P<0.0001; seed mass, P<0.0001) and larger plants above ground (stem length, P<0.0001) than did serpentine destination soil (Table 1). Notably, destination soils did not differ in their main effect on below ground traits (Table 1). Plants growing in serpentine destination soil therefore had proportionally greater allocation to root mass and symbiotic traits than plants growing in non-serpentine soil. While non-serpentine plant ecotypes experienced a greater proportional reduction in reproduction on serpentine vs. non-serpentine destination soil than did serpentine ecotypes (20% vs. 12% reduction in pod number), both ecotypes experienced lower reproduction in serpentine soil than in non-serpentine soil (Fig. 1a), though perhaps only in the presence of rhizobia (see section 4 of the Results below).

Because plant lineages used in this experiment were collected as seeds directly from the field, plant responses could be due to both genetic and maternal environmental effects. By conducting all statistical analyses as ANCOVAs and using initial seed weight as a covariate, the effects of maternal investment in seed weight were accounted for in the statistical model. Despite this precaution, plants from non-serpentine origin soil were larger vegetatively (stem length, P<0.0001; root mass, P<0.025) and had higher reproductive output at harvest (seed mass, P<0.01), detected as a main effect across treatments, which could reflect heritable ecotypic differences (Table 1). The covariate, initial seed weight, did not significantly affect any plant reproductive or vegetative traits (Table 1), although plants starting from larger seeds had slightly more nodules (P<0.025; data not shown). Results of the models had congruent trends regardless of whether initial seed size was included as a covariate.

### 2) Plant adaptive divergence in response to rhizobium ecotypes

*Medicago polymorpha* showed a general pattern of higher fitness when paired with rhizobia from its native soil type, and this effect did not depend on the soil environment (pod number, P<0.01) (Table 1, Fig. 1c). Least squares mean comparisons showed that when inoculated with non-serpentine rhizobia, non-serpentine plant ecotypes produced significantly more pods than did serpentine ecotypes, (P<0.001); when inoculated with serpentine rhizobia, serpentine plant ecotypes had a pattern of higher pod output than did non-serpentine ecotypes, although this latter effect was not significant (Fig. 1c). These crossing reaction norms suggest that selection in each soil type may have favored plant genotypes that could associate with rhizobia originating from that soil type. Interestingly, symbiotic trait values were greater with non-serpentine rhizobia than with serpentine rhizobia (nodule number: P<0.025; nodule mass: P<0.01; Table 1), though differing effects of rhizobium soil origin type were not detected for reproductive or vegetative plant traits. This pattern was driven by greater belowground allocation to nodules in plants inoculated with non-serpentine as opposed to serpentine rhizobia ((nodule mass)/(root mass); F_1,,8.25_ = 15.95, P<0.01).

### 3) Rhizobium fitnesss in symbiosis

Rhizobium symbiotic fitness estimates depended strongly on whether the plant host was grown in its native soil type (Table 1, Fig. 1b). The ANCOVA conducted on rhizobia fitness traits did not detect significant main effects of destination soil type, plant host ecotype, or rhizobial isolate (rhizobia ID). Rhizobia from non-serpentine soils formed more nodules (P<0.025) and more nodule biomass (P<0.01) than did rhizobia from serpentine soils (Table 1).

### 4) Importance of mutualism to the expression of plant traits

**Figure 3:**
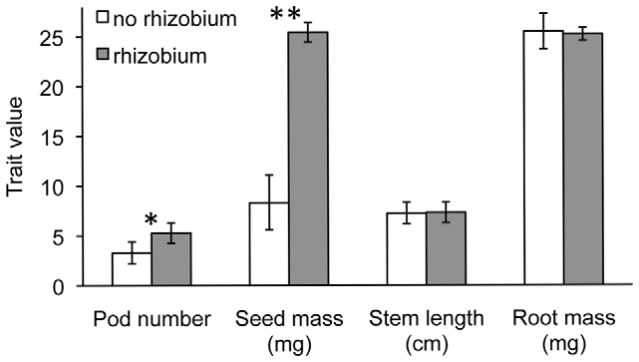
Effect of rhizobia on the expression of plant traits. Least square (LS) mean values from the simplified ANCOVA model for two reproductive traits, pod number and seed mass, and for two vegetative traits, stem length and root mass. Whilte columns indicate plants grown with no rhizobium (n = 70) and grey columns indicate plants grown with rhizobial inoculation (n = 403). Pod number and stem length and root mass were natural log transformed in the ANCOVA while seed mass and root mass means were calculated in a weighted ANCOVA; untransformed values are shown here. *P<0.001, 60% increase with rhizobium; **P<0.0001, 206% increase with rhizobium.

Rhizobia benefited plant reproduction but did not alter vegetative traits. A simplified ANCOVA model including both inoculated and uninoculated plants showed a 69% increase in seed mass (P<0.0001) and a 39% increase in pod number (P<0.001) in the presence of rhizobia (Table 2, Fig. 3). In contrast, the presence of rhizobia did not appear to affect plant size (Fig. 3).

**Table 2:** Mixed model ANCOVA for *M. polymorpha* grown either in the presence or absence of *E. medicae* in non-serpentine or serpentine destination soils in the greenhouse^1^.

MIXED MODEL ANCOVA			F-STATISTICS			
	df		Reproductive traits		Vegetative traits	
Fixed effects of interest	n	d	Ln pod num	Seed mass	Ln stem length	Root mass
R presence	1	14.3	**21.36** *c*	**34.5** *d*	0.01	0.02
Dest soil * R presence	1	12.1	1.6	**9.01** *b*	0.45	0.91
P origin * R presence	1	21.0	0.81	2.37	0.86	0.02

1 Only the fixed effects or interactions involving presence/absence of rhizobium are shown; random effects reflecting the main plot, split-plot and split-split-plot levels of organization of the factorial experimental design were not of experimental interest. Bonferroni correction for two hypotheses per test yields alpha = 0.025 as a threshold for significance; F-statistics in bold are significant. **†,** P<0.05; *b,* P<0.01; *c,* P<0.001; *d*, P<0.0001. Abbreviations: P, plant; R, rhizobium; df, degrees of freedom; n, numerator; d, mean denominator; Ln, natural log transform; num, number.

**Figure 4:**
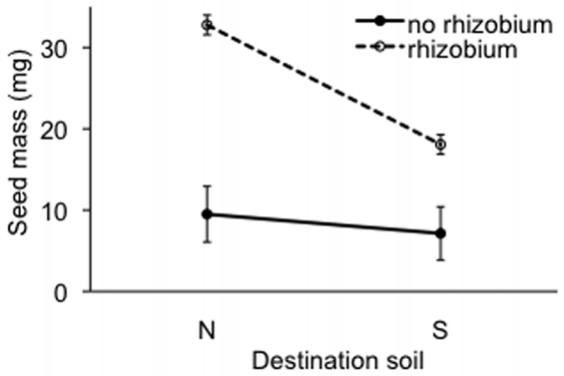
Context dependence of the impact of rhizobia on plant fitness. Reaction norm plot of total seed mass for plants grown with or without rhizobium in non-serpentine (N) and serpentine (S) destination soil, from the simplified weighted ANCOVA model. Bars are +/− LSM standard error.

The presence of rhizobia more strongly enhanced reproductive output on non-serpentine soil than on serpentine soil (interaction between rhizobium presence and soil type for seed mass: P<0.01; Table 2). LSmeans comparisons revealed an 84% increase in seed mass due to rhizobia on non-serpentine soil (P<0.0001), but only a 52% increase in seed mass on serpentine soil (P<0.001) (Fig. 4). LSmeans comparisons also revealed that seed mass was no different between non-serpentine and serpentine soils (P = 0.3804) in the absence of rhizobia. However, in the presence of rhizobia, there was a 35% increase in seed mass in non-serpentine relative to serpentine soil (P<0.0001) (Fig. 4).

### 5) Diversity of rhizobium inocula

Diversity in the inoculum had no significant effects on plant reproduction or vegetative traits, and affected only nodule mass from the symbiotic traits (P<0.025) (Table 1). Least squares mean comparisons revealed that for plants inoculated with non-serpentine rhizobia, nodule mass was marginally higher with single than with mixed inoculum (P<0.025), while for plants inoculated with serpentine rhizobia, nodule mass was higher with a mixed inoculum than with a single isolate (P<0.025).

## Discussion

### Plant ecotypes

The exotic legume, *Medicago polymorpha*, appears to have undergone adaptive divergence into ecotypes that invest more strongly in the mutualism with co-invading rhizobia in their home soil environment than they do in the contrasting soil type. Plants from serpentine and non-serpentine origins displayed different norms of reaction for fitness across the two destination soil types, suggesting that soil-based ecotypic differentiation has occurred. Results suggest that genetic divergence underlies these differences. Plant ecotypes matched to their home soil tended to have higher fitness than did mis-matched ecotypes, but this pattern was only significant for non-serpentine ecotypes. Therefore, this pattern partially fulfills expectations for local adaptation of ecotypes [2]. Notably, both plant ecotypes had greater total nodule biomass when matched to their native soil than did those plants mis-matched with soil type. The ability to form high nodule biomass in a home soil type could be a causal factor contributing to the vigor of plants in their home soil type. Larger biomass per nodule has been correlated with enhanced nutritional benefit to the plant ([52], [53], [54]; but see [48]), so greater total nodule biomass and nodule number in the home soil could represent a greater ability to acquire symbiotic nitrogen in this habitat. Greater nodule biomass in the native soil was not merely a by-product of greater plant vigor in the home soil. Greater allocation to symbiotic tissue in the home soil environment remained even when allocation to nodule biomass was measured relative to allocation to root biomass. Plants display greater allocation to the mutualism with rhizobia in their home soil, and greater allocation was correlated with enhanced reproductive output overall. The correlation between nodule biomass and reproductive output in this experiment was driven by effects of non-serpentine destination soil and less so by serpentine destination soil. However, greater nodule mass in serpentine soil might translate into a fitness advantage in nature (for example in the presence of competition or herbivory), though this fitness advantage was not detected in the greenhouse. To our knowledge, *M. polymorpha* does not inhabit serpentine soils in its native distribution. Assuming invasive plant lineages do not have an evolutionary history on serpentine in their native range, ecotypic divergence in this invasive species over the course of ∼150 years is striking, but not unanticipated, given that other invasive plants have formed similar ecotypes in response to serpentine [4]. However, this study highlights the point that the ability to support and allocate to a robust microbial symbiosis in a specific soil context could be a key adaptation in populations adapting to novel or heterogeneous environments during invasion.

While serpentine soil was a harsh habitat for both *M. polymorpha* ecotypes, it elicited ecotype-specific phenotypic responses. As would be expected for a physiologically stressful environment, reproductive output and plant height were reduced on serpentine relative to non-serpentine destination soil for both plant ecotypes. However, symbiotic serpentine ecotypes exhibited a less pronounced reduction in reproductive output on serpentine than did non-serpentine ecotypes. Furthermore, investment in nodules and roots revealed contrasting responses to soil type. Serpentine ecotypes showed an increase in below-ground investment in serpentine soil, relative to non-serpentine soil, whereas non-serpentine ecotypes showed a decrease. Below-ground traits are often sensitive to the ionic composition of the soil and robust below-ground investment have been utilized as a measure of serpentine tolerance [6], [55]. Tolerance of serpentine soil in *M. polymorpha* may depend upon the ability to plastically increase belowground allocation, especially to nodules.

### Interactions with rhizobium ecotypes

In addition to an ecotype-specific response to the abiotic component of soil environments, *M. polymorpha* ecotypes respond differently to rhizobia from the two soil types. The pattern of the plant-rhizobium ecotype interaction is in the direction of local adaptation by plants to soil-specific ecotypes of rhizobia. Plant ecotypes obtain greater fitness with rhizobia originating from a matched soil type; this difference is significant only for non-serpentine plant ecotypes and suggests adaptive divergence, but does not fulfill a strict test for local adaptation [2]. Overall, non-serpentine rhizobium ecotypes also induced higher nodule numbers and nodule biomass than did serpentine rhizobia. Since nodule number and biomass likely represent an important component of fitness for rhizobia [47], [48], these data suggest that some aspect of adaptation to serpentine soils could trade-off with the ability to gain high fitness on the host plant for serpentine rhizobia. Intriguingly, while plant ecotypes have differential fitness with rhizobium ecotypes, we did not detect a difference in the fitness benefits rhizobia received from different plant ecotypes. The results of this study support the more general finding that, while rhizobia may form soil ecotypes, these ecotypes may not offer a locally adaptive benefit to legume hosts across soil types, despite strong potential for rhizobial adaptation to soil in the free-living stage [56]. This observation is in contrast to that found for some mycorrhizae [21], [57], [58]. However, the support for rhizobium ecotypes is not as robust as that for plant ecotypes in this study because for each soil type, many fewer rhizobium isolates (2 in single isolate inocula and 9 in a mixed isolate inoculum) were utilized than were plant lineages (>75). Further experimental work manipulating a greater number of rhizobium isolates would be beneficial in clarifying these findings. This study included rhizobium inocula that were either mixed or single isolates, but inoculum diversity had only weak effects on plant responses, as has been found in other studies [47], [54].

The presence of rhizobia had strong effects on plant reproductive capacity, but weak effects on above- or belowground growth at maturity. Plants inoculated with rhizobia had strongly enhanced seed and pod output, relative to uninoculated plants, but experienced no enhancement of stem length or root mass. Because reproductive structures are the primary nitrogen sink in a legume at maturity, the additional nitrogen provided by rhizobia directly influences production of these tissues [59], [60]. Despite low levels of nitrogen present in the natural soils used in this experiment, plants were able to form vegetative structures, even in the absence of rhizobia. However, at reproduction a lack of rhizobia greatly reduced the number and mass of plant propagules. Studies that measure vegetative traits alone may miss important effects of soil mutualists if the appropriate classes of plant traits are not measured.

The benefits of nutritive soil mutualists to plants are thought to decrease in more nutrient-rich environments [61], [62], [63]. However, even though non-serpentine soil is a more nitrogen-rich, low stress environment, rhizobia conferred a greater benefit to host reproduction in non-serpentine relative to serpentine soil. This result supports the pattern observed by Thrall et al. (2008) [56], at the interspecific level in *Acacia*, for the evolution of increased strength of the rhizobia-legume mutualism in a low-stress environment, despite theoretical predictions to the contrary [19]. The abiotic stressors such as low calcium and heavy metal toxicity concomitant with low nitrogen in serpentine soil may reduce the capacity of a plant to derive benefits from rhizobia. Additionally, *E. medicae* may not have fully adapted to serpentine soil and this could reduce the benefits it confers upon *M. polymorpha* in the serpentine environment.

### Mutualism in co-invasion

Many invasive plants have reduced dependence on mycorrhizae which suggests that the dependence on effective symbiosis with soil microbes can be a limiting factor in invasions (reviewed in [13]). Although symbiosis with rhizobia confers a tremendous enhancement of reproductive output, the effects of symbiosis have not been as widely studied in invasive rhizobium-legume symbiosis as in invasive mycorrhizae-plant symbioses (but see [32], [33], [34], [35], [64], [65], [66]). Studies of plant-microbe mutualisms during invasion have often focused on plants that are broad generalists in terms of their symbiotic interactions with mycorrhizae [67], [68], although striking examples of specialists exist. For example, pine invasions of novel communities was facilitated by the co-introduction of appropriate ectomycorrhizae [23], [69]. *Medicago polymorpha* associates with the same symbiont species in both its native and invaded range, and so it is highly likely these two species have co-invaded California. Here, we provide one of the first experiments to document the importance of mutualism to adaptive divergence for a highly specific plant-microbe symbiosis. Adaptive divergence in plants for the soil-specific ability to invest in a robust mutualism may underlie the ability to colonize a novel heterogeneous environment.

The spread of exotic nitrogen-fixing plants can increase available soil nitrogen, which is often a limiting macronutrient. These exotics can facilitate the establishment of other invasive species and allow them to out-compete natives where nitrogen becomes abundant [25], [70], [71] and in extreme cases this can alter whole ecosystem function [72], [73]. *Medicago polymorpha* is considered a valuable pasture species because it increases available soil nitrogen [74], [75]. The adaptation of *M. polymorpha* to serpentine and subsequent increase in nitrogen deposition in a low nitrogen soil could act synergistically with other factors to facilitate further invasions of serpentine habitats that support a highly diverse endemic flora.

## Supporting Information

Figure S1 Map of locations within the McLaughlin Reserve where *M. polymorpha* and *E. medicae* genotypes and field soils were collected. Red squares are sites of serpentine genotype collections (S1, S2, S3, S4) and soil collection (S-soil); blue squares are sites of non-serpentine genotype collections (N1–N3) and soil collections (N-soil). Yellow line indicates the primary access road.
(TIF)

Figure S2 The split-split plot experimental design (n = 416 symbiotic plants, n = 72 rhizobium-free plants). Serpentine genotypes and soil are indicated by light grey and non-serpentine genotypes and soil are indicated by dark grey. Rhizobium treatment was applied as a main plot at the level of a rack; color around rack indicates the soil type from which rhizobium isolates were collected (16 rhizobium main plots for symbiotic plants, 2 for rhizobium-free plants). The destination soil sub-plot was applied at the level of a half-rack; color within rack indicates the soil type plants were grown in (32 destination soil sub-plots for symbiotic plants, 4 for rhizobium-free plants). The plant origin sub-sub-plot was applied within half-racks; color within circles indicates the soil type from which plant genotypes were collected. Therefore the weighted average for each sub-sub-plot (ie. a group of 6 or 7 plants) is the value for the most basic experimental unit in this hierarchical design (64 plant origin sub-sub-plots for symbiotic plants, 8 for rhizobium-free plants). N-rhizobia and S-rhizobia indicate groupings of rhizobium treatments that are comprised of rhizobia from non-serpentine or serpentine soils. Mix of 9, indicates rhizobium treatments comprised of a mix of 9 isolates of rhizobia; 1 and 2 indicate two different single isolate rhizobium treatments. Circles containing numbers indicate individual plants from serpentine soil (S1, S2, S3, S4) and non-serpentine soil (N1–N3) populations. The position of racks and the position of plants within half racks were completely randomized in the experiment.
(TIF)

Table S1 GPS coordinates of locations where *M. polymorpha* and *E. medicae* genotypes and field soils were collected.
(DOC)

Table S2 Experimental units and replication for main effects of interest from the ANCOVA analyses. For interactive effects, the experimental units for the lower level factor were utilized.
(DOCX)
