# Supplementary figures and images for: Mutualism and Adaptive Divergence: Co-Invasion of a Heterogeneous Grassland by an Exotic Legume-Rhizobium Symbiosis
Source: PLoS One. 2011 Dec 9;6(12):e27935. doi: 10.1371/journal.pone.0027935 (PMC3235091; doi:10.1371/journal.pone.0027935)

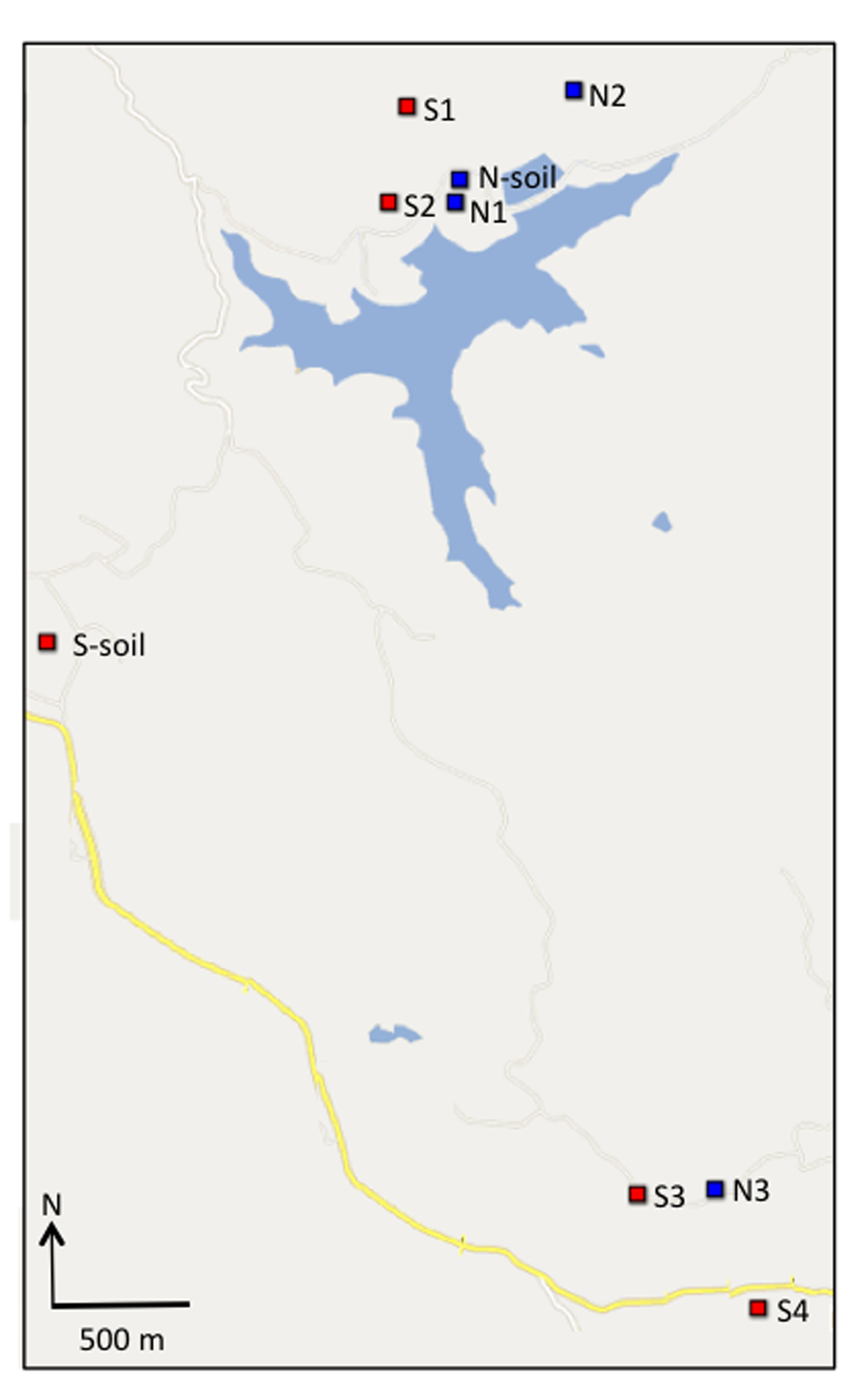

Supplement: Figure S1 — Map of locations within the McLaughlin Reserve where M. polymorpha and E. medicae genotypes and field soils were collected. Red squares are sites of serpentine genotype collections (S1, S2, S3, S4) and soil collection (S-soil); blue squares are sites of non-serpentine genotype collections (N1–N3) and soil collections (N-soil). Yellow line indicates the primary access road. (TIF) [file pone.0027935.s001.tif]

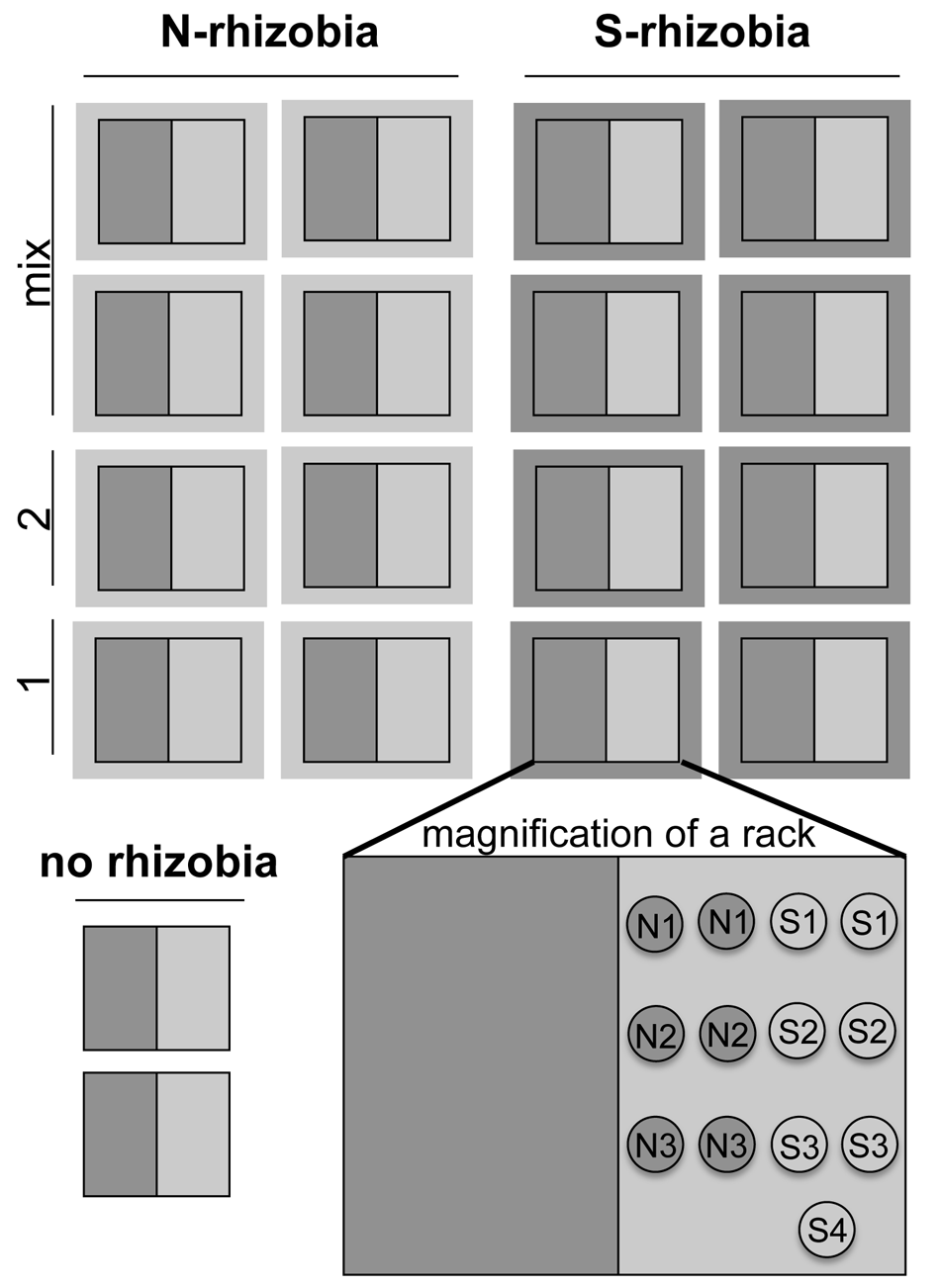

Supplement: Figure S2 — The split-split plot experimental design (n = 416 symbiotic plants, n = 72 rhizobium-free plants). Serpentine genotypes and soil are indicated by light grey and non-serpentine genotypes and soil are indicated by dark grey. Rhizobium treatment was applied as a main plot at the level of a rack; color around rack indicates the soil type from which rhizobium isolates were collected (16 rhizobium main plots for symbiotic plants, 2 for rhizobium-free plants). The destination soil sub-plot was applied at the level of a half-rack; color within rack indicates the soil type plants were grown in (32 destination soil sub-plots for symbiotic plants, 4 for rhizobium-free plants). The plant origin sub-sub-plot was applied within half-racks; color within circles indicates the soil type from which plant genotypes were collected. Therefore the weighted average for each sub-sub-plot (ie. a group of 6 or 7 plants) is the value for the most basic experimental unit in this hierarchical design (64 plant origin sub-sub-plots for symbiotic plants, 8 for rhizobium-free plants). N-rhizobia and S-rhizobia indicate groupings of rhizobium treatments that are comprised of rhizobia from non-serpentine or serpentine soils. Mix of 9, indicates rhizobium treatments comprised of a mix of 9 isolates of rhizobia; 1 and 2 indicate two different single isolate rhizobium treatments. Circles containing numbers indicate individual plants from serpentine soil (S1, S2, S3, S4) and non-serpentine soil (N1–N3) populations. The position of racks and the position of plants within half racks were completely randomized in the experiment. (TIF) [file pone.0027935.s002.tif]
