# Supplementary material for: Mutualism and Adaptive Divergence: Co-Invasion of a Heterogeneous Grassland by an Exotic Legume-Rhizobium Symbiosis
Source: PLoS One. 2011 Dec 9;6(12):e27935. doi: 10.1371/journal.pone.0027935 (PMC3235091; doi:10.1371/journal.pone.0027935)
Supplement: Table S1 — GPS coordinates of locations where M. polymorpha and E. medicae genotypes and field soils were collected. (DOC) [file pone.0027935.s003.doc]

| **Site** | **Coordinates** |
| --- | --- |
| N1 | 38.862631928,-122.358795353 |
| N2 | 38.866547555,-122.353409076 |
| N3 | 38.8279555875,-122.347122001 |
| S1 | 32,38.866017733,-122.360989914 |
| S2 | 38.862616112,-122.361796277 |
| S3 | 38.8278302361,-122.350638335 |
| S4 | 38.823817298,-122.345135075 |
| N-soil | 38.863468,-122.35858 |
| S-soil | 38.847169602,-122.377202915 |
