# Supplementary material for: Mutualism and Adaptive Divergence: Co-Invasion of a Heterogeneous Grassland by an Exotic Legume-Rhizobium Symbiosis
Source: PLoS One. 2011 Dec 9;6(12):e27935. doi: 10.1371/journal.pone.0027935 (PMC3235091; doi:10.1371/journal.pone.0027935)
Supplement: Table S2 — Experimental units and replication for main effects of interest from the ANCOVA analyses. For interactive effects, the experimental units for the lower level factor were utilized. (DOCX) [file pone.0027935.s004.docx]

| **ANCOVA model** | **Treatment** | **Experimental unit** | **n** |
| --- | --- | --- | --- |
| Symbiotic plants only | Plant origin | split half rack | 64 |
|  | Destination soil | half rack | 32 |
|  | Rhiz origin | rack | 16 |
|  | Rhiz div | rack | 16 |
|  | Rhiz ID | rack | 16 |
| Symbiotic and non-symbiotic plants | Rhiz presence | rack | 18 |
|  | Plant origin | split half rack | 72 |
|  | Destination soil | half rack | 36 |
